# The Anti-Apoptotic Effect of C-Type Natriuretic Peptide and the Regulation of *NPPC* in Porcine Ovarian Granulosa Cells

**DOI:** 10.3390/ijms262010046

**Published:** 2025-10-15

**Authors:** Xingyuan Liu, Jinlun Lu, Junyi Zeng, Lei An, Jianhui Tian, Guangyin Xi

**Affiliations:** Key Laboratory of Animal Genetics, Breeding and Reproduction of the Ministry of Agriculture and Rural Affairs, Frontiers Science Center for Molecular Design Breeding (MOE), College of Animal Science and Technology, China Agricultural University, Beijing 100193, China; 19806395675@163.com (X.L.); lujinlun0347@163.com (J.L.); z5408233@outlook.com (J.Z.); anleim@cau.edu.cn (L.A.)

**Keywords:** *NPPC*, *NPR2*, pig, ovarian granulosa cells, oocyte, tristetraprolin

## Abstract

C-type natriuretic peptide (CNP), encoded by the *NPPC* (Natriuretic Peptide Precursor C), has been recognized as the principal endogenous factor sustaining oocyte meiotic arrest in mammalian follicles. Yet its influence on porcine ovarian granulosa cell fate and the regulatory mechanism of *NPPC* expression within these cells remain poorly understood. Here, utilizing an in vitro culture model of primary porcine ovarian granulosa cells and immature oocytes, we examined the impact of CNP on granulosa cell apoptosis and oocyte meiotic resumption, and elucidated the molecular circuitry governing *NPPC* expression. We found that follicular atresia in pigs was accompanied by a marked decline in the CNP receptor NPR2 (natriuretic peptide receptor 2). Correspondingly, exogenous CNP suppressed apoptosis in cultured porcine granulosa cells. Estradiol can significantly promote the expression level of *NPPC* in porcine ovarian granulosa cells and, by enhancing NPR2 levels, it can synergize with CNP to inhibit oocyte meiotic resumption in vitro. Conversely, EGF signaling can significantly downregulate *NPPC* mRNA expression in porcine granulosa cells, an effect likely mediated by ERK-activated tristetraprolin (TTP). Collectively, these findings broaden our understanding of CNP in follicular development and delineate the endocrine network that controls *NPPC* transcription in the porcine ovary.

## 1. Introduction

C-type natriuretic peptide (CNP) is member of the natriuretic peptide family that is broadly distributed across mammalian tissues, including kidney, brain, heart, and vasculature, and is abundantly expressed in the ovary [1,2]. Within the follicle, CNP is synthesized and secreted by mural granulosa cells into the follicular fluid, where it acts in a paracrine manner to sculpt the microenvironment surrounding the oocyte. Its principal receptor, natriuretic peptide receptor 2 (NPR2), exhibits the highest affinity for CNP and is a transmembrane guanylyl cyclase. NPR2 is selectively localized to cumulus cells that encircle the oocyte. Upon CNP binding, NPR2 catalyzes the intracellular accumulation of cyclic guanosine monophosphate (cGMP) in cumulus cells; cGMP then traverses gap junctions into the oocyte, inhibits phosphodiesterase 3A (PDE3A), and thereby prevents the hydrolysis of cyclic adenosine monophosphate (cAMP). The resulting elevation in intra-oocyte cAMP arrests meiosis at the germinal vesicle (GV) stage [3]. This exquisite spatial compartmentalization creates a finely tuned intra-follicular communication network that positions CNP as a “natural brake” on oocyte maturation, a role that has come into sharp focus only within the past decade. Exploiting this mechanism, the natural factor synchronized in vitro oocyte maturation (NFSOM) protocol has been developed [4]. By supplementing culture media with CNP, NFSOM deliberately prolongs meiotic arrest in vitro, allowing additional time for the cytoplasmic and nuclear maturation events required for high-quality oocytes. The efficacy of this approach has been confirmed in cattle, sheep, goat, pig, horse, cat, and human [4,5,6,7,8,9,10]. Furthermore, CNP expression declines in ovaries of aged individuals; exogenous CNP supplementation restores mitochondrial function and markedly improves oocyte in vitro maturation (IVM) competence, underscoring its translational potential for enhancing fertility in advanced reproductive age [11,12].

In the ovary, paracrine and autocrine factors, together with pituitary gonadotropins, constitute a multidimensional regulatory network that precisely dictates the spatiotemporal expression and activity of the CNP/NPR2 system, thereby ensuring coordinated follicular development and oocyte maturation. Follicle stimulating hormone (FSH) upregulates *NPPC* and *NPR2* expression via the cAMP–PKA pathway. This effect is partially estrogen-dependent: FSH stimulates granulosa cells to synthesize estradiol, which in turn exerts positive feedback on *NPPC* and *NPR2* transcription [13]. Conversely, the pre-ovulatory luteinizing hormone (LH) surge suppresses the system. LH activates the epidermal growth factor receptor (EGFR) cascade, rapidly attenuating NPR2 guanylate cyclase activity and downregulating *NPPC* and *NPR2* mRNA [14,15]. TGF-β markedly increases *NPPC* mRNA and protein levels in cultured murine granulosa cells through SMAD3 binding to the *Nppc* promoter [16]. Oocyte-secreted factors (OSFs), including growth differentiation factor 9 (GDF9) and bone morphogenetic protein 15 (BMP15), enhance granulosa cell *NPR2* expression and amplify CNP/NPR2 signaling efficiency [17]. Although CNP and NPR2 are abundantly expressed in porcine granulosa cells and participate in meiotic regulation, the gonadotropic regulation of *NPPC* and *NPR2* expression in the porcine ovary remains largely unexplored.

Granulosa cell apoptosis is now recognized as the pivotal event that initiates follicle atresia and is governed by a multilayered, highly coordinated network of transcription factors, non-coding RNAs, hormones, and cytokines. FSH constitutes the principal survival factor for the dominant follicle. By activating the PKA-PI3K-AKT-FoxO1 axis, FSH suppresses FoxO1-mediated transcription of pro-apoptotic Bim and other death-promoting genes [18]. Concurrently, FSH stimulates granulosa cells to secrete local survival factors, including IGF-1 and VEGF, that act synergistically to suppress apoptosis [19,20]. Estrogen and FSH cooperate to enhance granulosa cell proliferation and viability. Estrogen, acting through estrogen receptors (ERα/ERβ), activates PI3K/AKT and MAPK/ERK pathways and attenuates the expression of apoptosis-related genes (e.g., *Bax*) [21,22]. Low concentrations of androgens serve as substrates for estrogen synthesis and, via androgen receptors (AR), promote granulosa cell proliferation and exert anti-apoptotic effects [23]. Growth factors such as EGF, TGF-α, and FGF engage their cognate receptors to activate PI3K/AKT and Ras/MEK/ERK cascades, thereby inhibiting apoptosis. In contrast, TNF-α, FasL, IFN-γ, and IL-1β are recognized as key pro-apoptotic mediators, and their expression is markedly elevated in atretic follicles [24,25]. Our previous work demonstrated that CNP mitigates DNA damage in in vitro cultured porcine cumulus–oocyte complexes [26]. The CNP/NPR2 system serves as a key regulator in shaping the follicular microenvironment. While previous studies have predominantly focused on its role in regulating meiotic progression in oocytes, the differential expression of *NPPC* and *NPR2* between healthy and atretic follicles, as well as the direct effects of CNP on granulosa cell apoptosis, remains to be fully elucidated. Clarifying these aspects will help expand our understanding of the broader functions of the CNP/NPR2 system in follicular development.

In this study, we aim to compare the expression levels of the *NPPC* and *NPR2* in granulosa cells from healthy and atretic follicles, further elucidate whether CNP affects apoptosis in porcine granulosa cells, and finally investigate the regulatory effects of estrogen and EGF signaling on the expression levels of *NPPC* and *NPR2* in porcine granulosa cells and their underlying mechanisms. We delineated the anti-apoptotic role of CNP in porcine granulosa cells and elucidated the gonadotropic regulation of *NPPC/NPR2* transcription. Our findings not only uncover a novel role for CNP in follicular development but also advance the current understanding of porcine folliculogenesis.

## 2. Results

### 2.1. Differential Expression of NPPC and NPR2 in Healthy and Atretic Porcine Follicles

**Figure 1:**
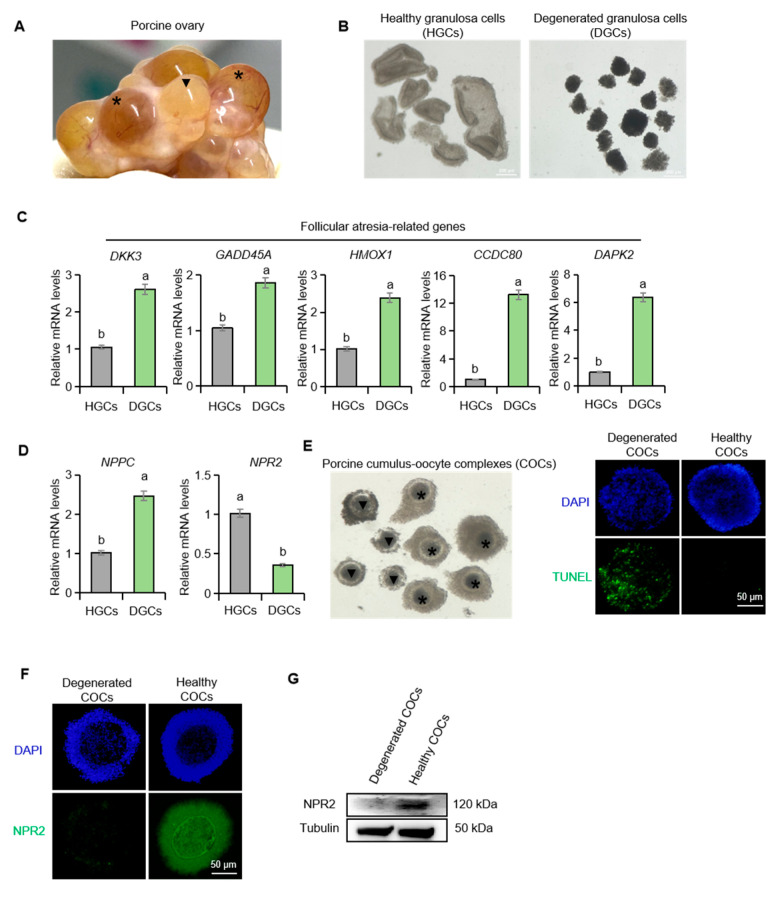
The expression of *NPPC* and *NPR2* in healthy and atretic porcine follicles. (**A**) The porcine ovary; the asterisk denotes healthy follicles, and the triangle denotes atretic follicles. (**B**) The healthy and degenerated mural granulosa cells isolated from porcine follicles. (**C**,**D**) The expression of follicular atresia-related genes: *NPPC* and *NPR2* in healthy and degenerated mural granulosa cells. (**E**) The healthy and degenerated COCs isolated from porcine follicles. (**F**,**G**) Immunofluorescence staining and Western blotting of NPR2 in healthy and degenerated porcine COCs. Values with superscript letters a and b are significantly different across columns according to Tukey’s test (*p* < 0.05), n = 6. *DKK3*, Dickkopf-related protein 3; *GADD45A*, growth arrest and DNA-damage-inducible alpha; *HMOX1*, Heme oxygenase 1; *CCDC80*, coiled-coil domain-containing 80; *DAPK2*, death-associated protein kinase 2.

To characterize *NPPC* and *NPR2* expression in healthy versus atretic follicles, mural granulosa cells were first isolated from porcine ovarian follicles. Healthy granulosa cells (HGCs) appeared intact and uniformly bright white, whereas degenerated granulosa cells (DGCs) were loosely arranged and appeared dark brown to black (Figure 1A,B). Quantitative real-time PCR (qPCR) confirmed the divergent cellular states. Transcripts of follicular atresia-associated genes (*DKK3*, *GADD45A*, *HMOX1*, *CCDC80*, *DAPK2*) were markedly elevated in DGCs (Figure 1C). This indicates that our sample collection is representative. Notably, *NPPC* mRNA was significantly upregulated, while *NPR2* mRNA was significantly downregulated in DGCs compared with HGCs (Figure 1D). We next compared NPR2 expression in cumulus–oocyte complexes (COCs) derived from healthy and atretic follicles. TUNEL staining effectively distinguished the two COCs populations (Figure 1E). Immunofluorescence and Western blotting revealed a pronounced reduction in NPR2 protein levels in degenerated COCs from atretic porcine follicles (Figure 1F,G).

### 2.2. CNP Suppresses Apoptosis in Porcine Granulosa Cells Cultured In Vitro

**Figure 2:**
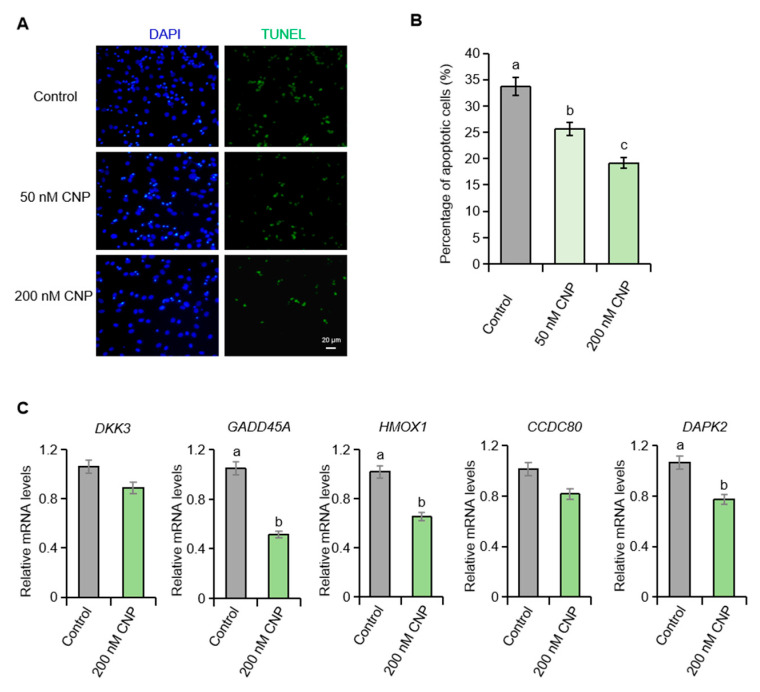
CNP suppresses apoptosis in porcine granulosa cells cultured in vitro. (**A**,**B**) TUNEL staining of apoptotic porcine granulosa cells in control and CNP-treated groups with statistical analysis. (**C**) The expression of follicular atresia-related genes in control and CNP-treated porcine granulosa cells. Values with superscript letters a, b and c are significantly different across columns according to Tukey’s test (*p* < 0.05), n = 4. *DKK3*, Dickkopf-related protein 3; *GADD45A*, growth arrest and DNA-damage-inducible alpha; *HMOX1*, Heme oxygenase 1; *CCDC80*, coiled-coil domain-containing 80; *DAPK2*, death-associated protein kinase 2.

The marked down-regulation of NPR2 in degenerated granulosa cells prompted us to hypothesize that the CNP/NPR2 axis participates in granulosa cell apoptosis in pigs. To test this, primary porcine ovarian granulosa cells were cultured in vitro and exposed to exogenous CNP. Consistent with our hypothesis, CNP treatment significantly reduced the apoptotic index (Figure 2A,B) and attenuated the expression of follicular atresia-associated genes (Figure 2C).

### 2.3. Estrogen Upregulates the mRNA Levels of NPPC and NPR2 in Porcine Granulosa Cells

**Figure 3:**
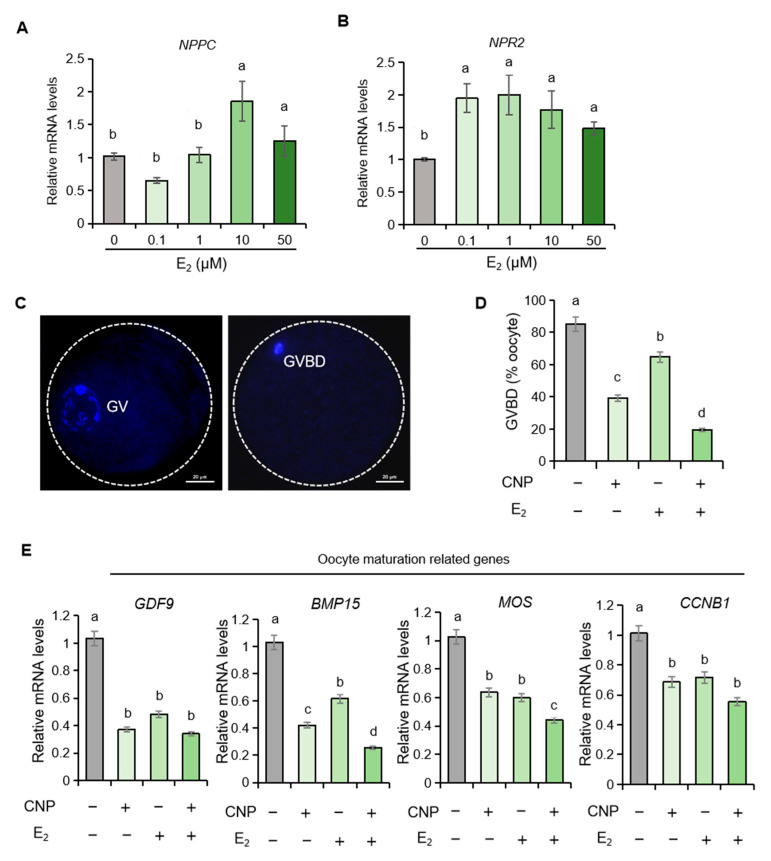
Estrogen synergizes with CNP to orchestrate meiotic resumption in porcine oocytes. (**A**,**B**) The expression of *NPPC* and *NPR2* mRNA in control and E_2_-treated porcine granulosa cells. (**C**,**D**) Representative DAPI staining of oocytes at the GV and GVBD stages, and analysis of GVBD rates in different treatment groups. (**E**) The expression of oocyte maturation-related genes in different treatment groups. CNP, 200 nM; E_2_, 100 nM. Values with superscript letters a, b, c, and d are significantly different across columns according to Tukey’s test (*p* < 0.05), n = 6. *GDF9*, growth differentiation factor 9; *BMP15*, bone morphogenetic protein 15; *MOS*, MOS proto-oncogene, serine/threonine kinase. *CCNB1*, cyclin B1.

Estrogen (E_2_) is a pivotal endocrine hormone within the follicular microenvironment. We therefore examined its influence on *NPPC* and *NPR2* expression in porcine granulosa cells. Treatment with estrogen elicited a pronounced, concentration-dependent up-regulation of both *NPPC* and *NPR2* transcripts (Figure 3A,B), implicating estrogen as a positive regulator of the CNP/NPR2 signaling axis in the porcine ovary.

### 2.4. Estrogen Synergizes with CNP to Orchestrate Meiotic Resumption in Porcine Oocytes

Building on estrogen’s ability to upregulate *NPR2*, we examined whether estrogen could potentiate CNP-dependent meiotic arrest in porcine oocytes. The results showed that the germinal vesicle breakdown (GVBD) rates were significantly lower in oocytes treated with CNP plus estrogen than in those treated with CNP alone (Figure 3C,D). Concordantly, qPCR revealed that estrogen amplified CNP’s suppressive effect on the pro-maturation genes *BMP15* and *MOS* (Figure 3E). These findings preliminarily demonstrate that estrogen acts synergistically with CNP to sustain meiotic arrest in pig oocytes.

### 2.5. EGF Suppresses the Expression of NPPC and NPR2 in Porcine Granulosa Cells

**Figure 4:**
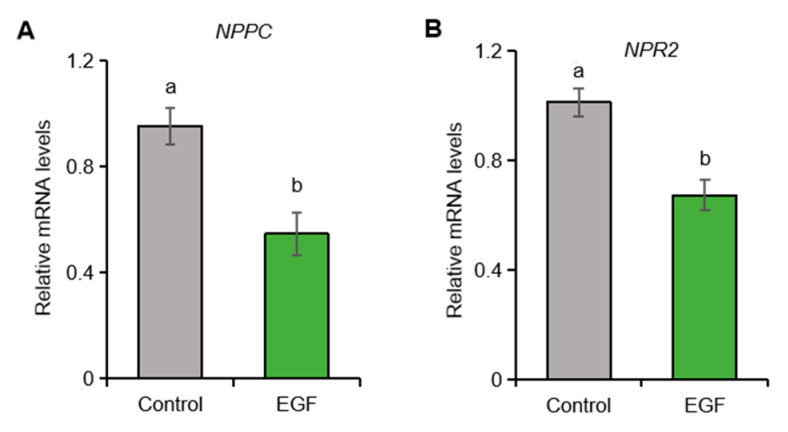
The effect of EGF on the expression of *NPPC* and *NPR2* in porcine granulosa cells. (**A**,**B**) The expression of *NPPC* and *NPR2* mRNA in control and EGF-treated porcine granulosa cells. Values with superscript letters a and b are significantly different across columns according to Tukey’s test (*p* < 0.05); n = 4.

Studies in mice indicate that the pre-ovulatory LH surge markedly reduces *NPPC* mRNA in granulosa cells, with EGF signaling serving as the primary downstream mediator [27]. We therefore examined whether EGF exerts a comparable effect in porcine granulosa cells. Exposure to EGF significantly suppressed *NPPC* and *NPR2* transcript abundance (Figure 4), confirming that EGF signaling represses the CNP/NPR2 system in the porcine ovary.

### 2.6. EGF Promotes ZFP36/TTP Expression in Porcine Granulosa Cells

**Figure 5:**
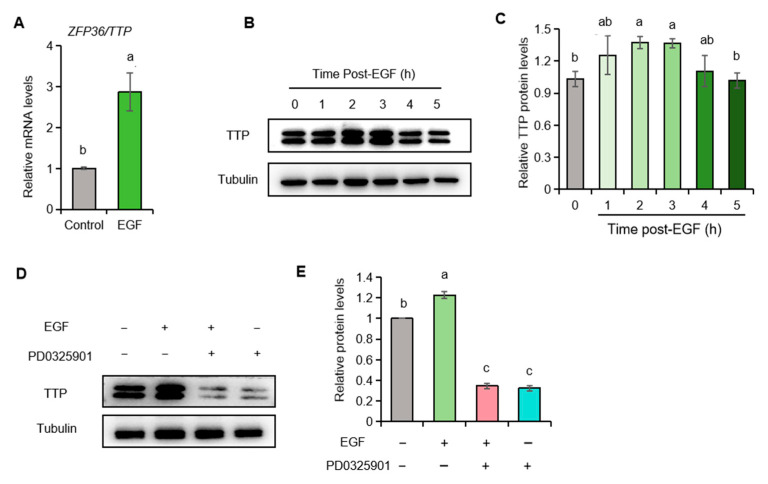
EGF modulates the expression of the RNA-binding protein TTP in porcine ovarian granulosa cells. (**A**) The expression of *ZFP36* mRNA in control and EGF-treated porcine granulosa cells. (**B**,**C**) Western blotting of TTP in porcine granulosa cells at different time points after EGF treatment. (**D**,**E**) Western blotting of TTP in different porcine granulosa cell treatment groups. Values with superscript letters a, b, and c are significantly different across columns according to Tukey’s test (*p* < 0.05); n = 3. *ZFP36*, *ZFP36* ring finger protein, the gene encoding TTP.

Our previous study demonstrated that the RNA-binding protein TTP degrades *NPPC* mRNA in murine granulosa cells [28]. We next explored the mechanism by which EGF reduces *NPPC* mRNA in porcine granulosa cells. We found that EGF treatment significantly increased both *ZFP36* mRNA and TTP protein (Figure 5A–C). Furthermore, we discovered that the MEK antagonist PD0325901 reversed the EGF-induced upregulation of TTP (Figure 5D,E), indicating that EGF upregulates TTP via the MEK pathway in porcine granulosa cells.

### 2.7. TTP Downregulates NPPC mRNA in Porcine Granulosa Cells

**Figure 6:**
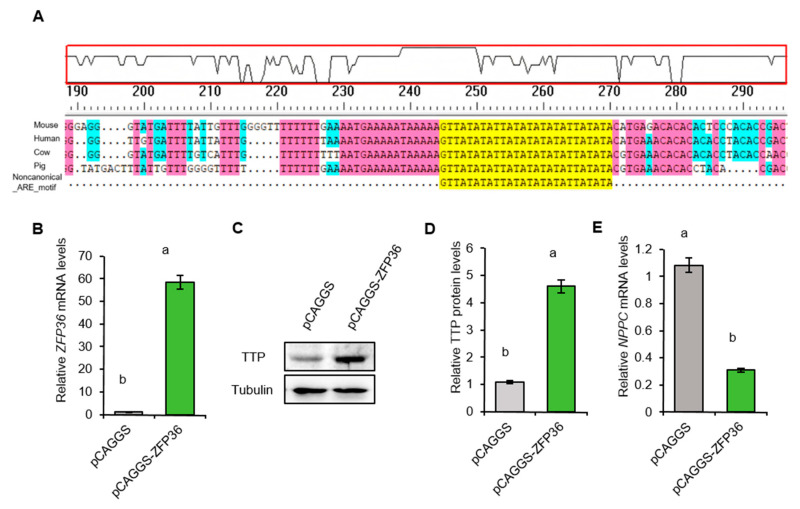
TTP downregulates *NPPC* mRNA in porcine ovarian granulosa cells. (**A**) Cross-species conservation analysis of non-canonical AU-rich element (ARE) motifs in *NPPC* mRNA sequences from mouse, human, cow, and pig. Blue indicates residues identical in at least three sequences, red in at least four, and yellow in all five sequences. (**B**) The expression of *ZFP36* mRNA in the control and gene overexpression group. (**C**,**D**) Western blotting of TTP in the control and *ZFP36* overexpression group. (**E**) The expression of *NPPC* mRNA in the control and *ZFP36* overexpression group. Values with superscript letters a and b are significantly different across columns according to Tukey’s test (*p* < 0.05); n = 3.

TTP initiates *NPPC* mRNA decay in mouse granulosa cells by binding to non-canonical AU-rich elements (AREs) within the 3′-untranslated regions (3′-UTRs) [28]. Comparative analysis revealed that these non-canonical AREs are highly conserved in the *NPPC* 3′-UTRs of mouse, human, cow, and pig species (Figure 6A). In functional validation, *ZFP36* overexpression in porcine granulosa cells markedly reduced *NPPC* mRNA levels (Figure 6B–E).

## 3. Discussion

The follicular microenvironment constitutes an exquisitely orchestrated and highly dynamic ecosystem that integrates cellular constituents (oocyte, granulosa, and theca cells), molecular cues (hormones, growth factors, and reactive oxygen species), and physical parameters (hypoxia and vascularization) [29]. Its homeostasis and functional quality are the primary determinants of oocyte competence, thereby dictating female fertility and the success rates of assisted reproductive technology. Over the past decade, CNP has emerged as a pivotal regulator of this microenvironment. Synthesized predominantly by mural granulosa cells and secreted into follicular fluid, CNP exerts paracrine control over the follicular microenvironment. Elucidating the molecular mechanisms governing CNP/NPR2 expression will therefore illuminate the precise paracrine and gap junctional dialogues between the oocyte and its surrounding somatic cells, and clarify how nuclear and cytoplasmic maturation are synchronized, an essential prerequisite for obtaining high-quality oocytes.

The apoptotic fate of granulosa cell is dictated by the balance between pro- and anti-apoptotic signals within the follicular microenvironment [25]. The down-regulation of NPR2 in granulosa cells of porcine atretic follicles likely attenuates CNP/NPR2 signaling within the follicle. Consistent with this interpretation, exogenous CNP markedly suppressed apoptosis in cultured porcine granulosa cells. Previous studies have shown that another pleiotropic peptide widely present in follicles, apelin, also has the dual effects of inhibiting granulosa cell apoptosis and promoting cGMP synthesis by stimulating the release of nitric oxide [30,31]. *Npr2* null mice exhibit defective cumulus oophorus formation and a striking loss of cumulus cells [32], a phenotype we postulate arises from *NPR2* deficiency-triggered apoptosis. In addition, previous studies have shown that cGMP supplementation significantly inhibits granulosa cell apoptosis in serum-free cultured mouse preantral follicles [33]. However, while the source of this protective cGMP remains undefined, our published data demonstrating that CNP promotes preantral follicle growth in vitro implicate the CNP/NPR2 system as a potential source [34]. Based on our findings and previous reports, we speculate that the decreased cGMP may contribute to the apoptosis in porcine granulosa cells. Intriguingly, degenerating porcine granulosa cells displayed a marked upregulation of *NPPC* mRNA. A mechanistic parallel has been reported in bovine coronary artery endothelial cells exposed to oxidative stress, where *NPPC* expression was significantly upregulated [35] Furthermore, given that oxidative stress is a well-known contributor to granulosa cell apoptosis and follicular atresia, we speculate that the increased expression of *NPPC* in granulosa cells of atretic follicles may act as a defensive strategy to resist apoptosis or adapt to the deteriorating follicular microenvironment. Collectively, our findings established the CNP/NPR2 pathway as a potential anti-apoptotic regulator of porcine granulosa cells; however, the precise downstream signaling cascades merit further investigation.

Estrogen is a pivotal endocrine hormone that sustains healthy folliculogenesis, stimulates granulosa cell activity, and suppresses follicular atresia. The capacity of estrogen to govern the CNP/NPR2 signaling axis has consequently attracted considerable attention. In mice, estrogen–estrogen receptor complexes directly engage estrogen responsive elements within the *NPPC* promoter to drive *NPPC* transcription [36], and our previous bovine studies revealed an analogous estrogenic induction of *NPPC* expression [4]. Here, we demonstrate that estrogen likewise upregulates both *NPPC* and *NPR2* in porcine granulosa cells, underscoring a highly conserved regulatory mechanism across mammalian species. Cultured porcine granulosa cells abundantly express estrogen receptors; future work should clarify whether the cis-acting elements and trans-acting factors that mediate estrogen-dependent *NPPC* transcription are similarly conserved. Beyond enhancing *NPPC* expression, estrogen also elevated *NPR2* levels in granulosa cells, augmenting CNP’s ability to prevent meiotic resumption in porcine oocytes. Combined treatment with CNP and estrogen maintained meiotic arrest more effectively than either agent alone. Given the expanding use of CNP priming prior to IVM to boost oocyte quality and developmental competence, our findings provide a mechanistic rationale for incorporating estrogen into such protocols. By optimizing CNP–estrogen synergy, IVM outcomes and overall efficiency of in vitro embryo production may be further improved.

The pre-ovulatory LH surge terminates the CNP/NPR2-mediated brake on meiotic resumption through EGF signaling, thereby launching nuclear maturation and enabling the production of fertilization-competent oocytes [15]. Here, we show that EGF markedly diminishes *NPPC* and *NPR2* transcript levels in porcine granulosa cells, mirroring earlier observations and underscoring a conserved mechanism by which LH signaling attenuates the CNP/NPR2 axis across mammalian species. We found EGF signaling robustly upregulated the TTP, a well-characterized mRNA-destabilizing factor [28], and overexpression of TTP significantly reduced *NPPC* mRNA levels in porcine granulosa cells. Previous studies have indicated that the overactivated CNP/NPR2 system in follicles is involved in the formation of polycystic ovary syndrome (PCOS), which is specifically manifested by the abnormal elevation of *Nppc/NPPC* and *Npr2*/*NPR2* in follicles of PCOS mouse models and human patients [37,38]. This elevation causes the blockage of oocyte meiotic resumption and anovulation. In our study, we found that TTP can significantly downregulate the expression of *NPPC* mRNA, which may provide a new target for future research and clinical treatment of PCOS. Collectively, these findings expand the endocrine/paracrine circuitry governing the CNP/NPR2 signaling system in porcine follicles, refining our understanding of the molecular choreography that orchestrates follicular development and intercellular communication.

The expression patterns and functions of the natriuretic peptide family in the ovary exhibit distinct species-specific characteristics. For instance, both CNP and BNP can inhibit the resumption of meiosis in porcine oocytes [39], whereas only CNP has this effect in bovine and mice [4,17]. Additionally, *NPR2* is prominently expressed in bovine oocytes but is only minimally expressed in porcine oocytes and completely absent in murine oocytes [4,17,26]. Therefore, some of the findings from our study merit further investigation, such as whether CNP also has an inhibitory effect on granulosa cell apoptosis in other species. Moreover, the interpretation of our results should be tempered by the recognition of the constraints associated with in vitro granulosa cell cultures. Our monolayer culture system does not recapitulate the critical paracrine interactions between granulosa cells, theca cells, and the oocyte, all of which are indispensable for normal follicular function and steroidogenesis in vivo. Furthermore, the hormonal milieu in vitro is static and oversimplified. In vivo, granulosa cells are exposed to dynamic, pulsatile secretions of gonadotropins within a specific vascular microenvironment, which we cannot fully replicate [40,41]. In the future, the downstream signaling pathways and key target genes through which CNP inhibits granulosa cell apoptosis can be thoroughly investigated across multiple species and cell models, such as animal models with granulosa cell-specific *NPPC* knockout and in vitro follicle culture. Additionally, whether the method of estrogen synergizing with CNP to maintain meiotic arrest can be applied to oocyte in vitro maturation systems to improve nuclear and cytoplasmic maturation synchrony, thereby enhancing the quality and developmental potential of in vitro matured oocytes, remains to be further explored. Finally, the identification of transcription factors that regulate TTP expression is also essential, as this will help reveal the molecular mechanisms underlying EGF signaling-induced TTP expression.

## 4. Materials and Methods

### 4.1. Isolation and Cultivation of Porcine Granulosa Cells

Porcine ovaries were procured from a local abattoir and transported to the laboratory within 2 h in pre-warmed (38 °C) sterile saline. Primary granulosa cells (GCs) were aspirated under sterile conditions from 3 to 6 mm antral follicles using a 10 mL syringe, and then washed three times in warm saline to remove excess blood. Cumulus–oocyte complexes (COCs) were discarded, and the remaining GCs were pelleted by low-speed centrifugation (1000 rpm, 5 min) and rinsed three additional times with PBS to eliminate residual white blood cells. The purified GCs were seeded into culture dishes and maintained in DMEM/F12 (11320033, Gibco, Grand Island, NY, USA) supplemented with 10% fetal bovine serum (10099141, Gibco, Grand Island, NY, USA) and 1% GlutaMAX™ (35050061, Gibco, Grand Island, NY, USA). Twenty-four hours post-isolation, cells were washed three times with PBS to remove any remaining non-adherent cells, and the medium was replaced by FBS-free DMEM/F12 with or without 200 nM CNP (N8768, Sigma-Aldrich, St. Louis, MO, USA), 100 nM estrogen (E1024, Sigma-Aldrich, St. Louis, MO, USA) and 20 ng/mL EGF (SRP3027, Sigma-Aldrich, St. Louis, MO, USA). Healthy and atretic antral follicles were classified under a stereomicroscope (SMZ18, Nikon Instruments, Melville, NY, USA), using vascularization of the follicular wall and the clarity of follicular fluid as primary criteria [34]. Healthy antral follicles exhibited a pinkish hue, a well-defined vascular sheath, and transparent follicular fluid, whereas atretic antral follicles appeared opaque, lacked discernible surface vessels, and contained abundant cellular debris. The accuracy of this morphological classification was subsequently validated by quantitative real-time PCR of atresia-associated gene markers.

### 4.2. Isolation and Cultivation of Porcine COCs

COCs were aspirated from 3 to 6 mm follicles using an 18-gauge needle attached to a 10 mL syringe. After three washes in HEPES-buffered TCM-199 (2340030, Gibco, Grand Island, NY, USA) supplemented with 0.8 mM L-glutamine (G8540, Sigma-Aldrich, St. Louis, MO, USA) and 3 mg/mL bovine serum albumin (A8806, Sigma-Aldrich, St. Louis, MO, USA), COCs were cultured in groups of 50–60 per 400 µL in four-well dishes containing TCM-199 with 3 mg/mL BSA, 0.8 mM L-glutamine, 75 µg/mL potassium penicillin G, and 50 µg/mL streptomycin sulfate. Each well contained 400 µL of this medium overlaid with 400 µL mineral oil and pre-equilibrated for at least 3 h at 38.5 °C in 5% CO_2_. Then, CNP, estradiol, or a CNP and estradiol combination was added to the culture medium as required by the experimental design.

### 4.3. RNA Extraction and Quantitative Real-Time RT-PCR

Total RNA from granulosa cells was isolated using TRIzol (15596018CN, Life Technologies, Carlsbad, CA, USA) following the manufacturer’s instructions. The amount of RNA was determined by calculating the ratio of absorbance at 260 nm to the absorbance at 280 nm. Using the PrimeScript RT reagent kit (RR037A, TaKaRa Bio, Kusatsu, Shiga, Japan), cDNA was produced after reverse transcription of RNA. The amplification was performed using iQ SYBR Green Supermix (1708880, Bio-Rad, Hercules, CA, USA). A standard curve using serial dilutions of a pooled sample (cDNA from all samples), a negative control without cDNA template, and a negative control without reverse transcriptase (RT) were included in every assay. Only standard curves with efficiency between 90% and 110% and a correlation coefficient above 0.99 were accepted. Data were normalized against the reference gene Rps18, which was chosen based on stable gene expression levels (geNorm; Ghent University Hospital, Ghent, Belgium). Primers were designed using the National Center for Biotechnology Information Primer Blast (http://www.ncbi.nlm.nih.gov, accessed on 24 February 2025). The primers used for each gene are summarized in Table S1. The experiments were performed at least three times with biological replicates.

### 4.4. Western Blot Analysis

The granulosa cells were sonicated (40% AMPL, 30 s pulse on, 30 s pulse off, 3 min) in RIPA Lysis Buffer (P0013B, Beyotime Biotechnology, Shanghai, China) containing 1 mmol/L phenylmethyl sulfonyl fluoride (PMSF). The proteins isolated from the samples were boiled in a loading buffer for 10 min, and the protein lysates (approximately 15 µg) were electrophoresed on 12% SDS-PAGE and then transferred onto polyvinylidene difluoride (PVDF) membranes (1620177, Bio-Rad Laboratories, Hercules, CA, USA; U = 20 V, 0.1 A). The anti-NPR2 antibody (1:1000 dilution; 55113-1-AP, Proteintech Group, Rosemont, IL, USA), anti-TTP antibody (1:1000 dilution; ab124024, Abcam, Cambridge, UK), and anti-Tubulin antibody (1:5000 dilution; ab18207, Abcam, Cambridge, UK) were incubated with the samples at 4 °C overnight. The membranes were then washed three times in Tris-buffered saline with Tween 20 (TBST) and incubated with horseradish peroxidase (HRP)-conjugated anti-rabbit or anti-mouse antibody. Analysis was performed with enhanced chemiluminescence detection reagents (Applygen Technologies, Inc., Beijing, China) according to the manufacturers’ instructions. Protein bands were analyzed with ImageJ software (Version 1.53t; National Institutes of Health, Bethesda, MD, USA).

### 4.5. Immunofluorescence

The COCs were initially fixed in 4% paraformaldehyde (PFA) (P0099, Beyotime Biotechnology, Shanghai, China) for 1 h, permeabilized with 0.5% PBST, and subsequently blocked with 1% (*w*/*v*) BSA in 0.5% PBST for 6 h. For NPR2 staining, the COCs were incubated overnight at 4 °C with specific antibodies (1:5000 dilution; A7906, Sigma-Aldrich, St. Louis, MO, USA). After three washes, the COCs were incubated with secondary antibodies (488 goat anti-mouse IgG; 1:500 dilution; A-11008, Thermo Fisher Scientific, Waltham, MA, USA) for 1 h, with the nuclei counterstained using DAPI. The fluorescence was visualized using an inverted epifluorescence microscope (IX71; Olympus, Tokyo, Japan). The experiments were conducted in triplicate biological replicates.

### 4.6. TdT-Mediated dUTP Nick-End Labeling (TUNEL) Assay

The apoptotic status of the granulosa cells and COCs was evaluated using a TUNEL assay kit (C1086, Beyotime Bio-technology, Shanghai, China). The granulosa cells and COCs were fixed with 4% PFA, permeabilized in 0.5% PBST for 20 min, and incubated in 0.5% BSA-PBS-PVA at room temperature. The granulosa cells and COCs were then incubated in the TUNEL mixture for 1 h at 37 °C in the dark. After staining, the granulosa cells and COCs were counterstained with DAPI. The labeled granulosa cells and COCs were mounted on glass slides and imaged. The apoptosis rate was calculated as follows: apoptosis rate = (the number of TUNEL-positive cells/total cell number) × 100%. ImageJ software was employed for analysis. The experiments were conducted in triplicate biological replicates.

### 4.7. Analysis of Nuclear Stages of Oocytes

After 24 h of in vitro culture of COCs, the oocytes were mechanically denuded. The oocytes were then fixed in 4% paraformaldehyde (PFA) for 1 h and permeabilized in 0.5% PBST (0.5% Triton-100 (*v*/*v*) in PBS containing 0.1% (*w*/*v*) polyvinyl alcohol (PVA)). They were then stained with DAPI for 5 min. The stained oocytes were placed on a glass slide, which was then covered with a coverslip. Images of the stained oocytes were captured using an inverted epifluorescence microscope (IX71; Olympus, Tokyo, Japan). The stained oocytes were then classified based on the morphologies of the chromatin and nuclear envelope [42]. The percentages of the oocytes at the GVBD stage in each experimental group were calculated.

### 4.8. Construction and Transfection of Overexpression Vectors

The coding sequence of porcine *ZFP36*/TTP gene (NM_001168419.1) was obtained from the cDNA of porcine ovarian granulosa cells using PrimeSTAR Max DNA polymerase (R045A, TaKaRa Bio, Kusatsu, Shiga, Japan) following the manufacturer’s instructions. The purified PCR product was then cloned into the PCAGGS-2A-EGFP vector. All recombinant plasmids were identified by sequencing. The primers used for PCR amplification are summarized in Table S1. Porcine granulosa cells were plated in a 12-well plate and cultured to 70% confluence after which 1.5 μg overexpression or empty vector was introduced into the cells using the lipo2000 transfection reagent (11668019, Invitrogen, Carlsbad, CA, USA) according to the manufacturer’s instructions. At 24 h after transfection, granulosa cells were washed with PBS twice and collected for subsequent experiments.

### 4.9. Statistics Analysis

In this study, statistical analyses were performed using SPSS 22.0 (SPSS, Chicago, IL, USA). The data were analyzed using a one-way analysis of variance (ANOVA) and differences between groups were assessed using Tukey’s test for multiple means comparison. All data are presented as means ± standard error of the mean (SEM). A *p*-value < 0.05 was considered statistically significant.

## 5. Conclusions

We found that NPR2 expression is markedly downregulated in porcine atretic follicles and that CNP exerts an anti-apoptotic effect on porcine granulosa cells. Estrogen further potentiates CNP signaling by up-regulating *NPR2*, thereby sustaining meiotic arrest in porcine oocytes. Moreover, in porcine ovarian granulosa cells, EGF signaling may downregulate *NPPC* expression by up-regulating TTP, as previously reported in mice and cattle. Collectively, these findings illuminate novel roles for CNP in porcine ovarian physiology and underscore its relevance to follicular development.

## Data Availability

The raw data supporting the conclusions of this article will be made available by the authors, without undue reservation.

## Supplementary Materials

The following supporting information can be downloaded at https://www.mdpi.com/article/10.3390/ijms262010046/s1.
